# *Nakaseomyces glabrata* endocarditis: A therapeutic dilemma

**DOI:** 10.1016/j.mmcr.2023.04.002

**Published:** 2023-04-28

**Authors:** Kin Ki Jim, Joelle J.N. Daems, S. Matthijs Boekholdt, Karin van Dijk

**Affiliations:** a Amsterdam UMC Location University of Amsterdam, Department of Medical Microbiology and Infection Prevention, Meibergdreef 9, Amsterdam, 1105 AZ, the Netherlands; b Amsterdam Institute for Infection and Immunity, De Boelelaan 1085, Amsterdam, 1081 HV, the Netherlands; c Amsterdam UMC Location University of Amsterdam, Department of Cardiology, Meibergdreef 9, Amsterdam, 1105 AZ, the Netherlands

**Keywords:** *Nakaseomyces glabrata*, *Candida glabrata*, Endocarditis, Invasive candidiasis, Anti-fungal therapy

## Abstract

*Candida* infective endocarditis is a rare but serious entity that often requires aggressive treatment. However, treatment can be challenging in patients infected with drug-resistant fungi and/or with substantial comorbidity. Moreover, recommendations in treatment guidelines for these patients are based on limited clinical data due to their rarity. Here we report a case of *Nakaseomyces glabrata* (*Candida glabrata*) prosthetic valve endocarditis in a patient with congenital heart disease. This case illustrates a therapeutic dilemma for *Nakaseomyces glabrata* prosthetic valve endocarditis and the need for novel antifungal drugs and further clinical studies.

## 1 Introduction

*Candida* infective endocarditis is a rare complication of candidiasis but is associated with a high disease burden [[1], [2], [3]]. *Nakaseomyces glabrata*, formerly known as *Candida glabrata*, is an opportunistic human fungal pathogen that can cause a wide range of infections, ranging from superficial infections (e.g., mucositis and soft tissue infections) to invasive infections, such as endocarditis [4,5]. It is one of the most prevalent causes of invasive candidiasis after *Candida albicans* [6,7]. Treatment of *N. glabrata* can be challenging because of its intrinsic resistance to azole antifungal therapy, and in recent years, an increase in the resistance rate against echinocandins has been observed [8,9]. In the recently published WHO fungal priority pathogen list to raise awareness for the emerging global health threat of invasive fungal infections and antifungal drug resistance, *N. glabrata* was categorized into the high-priority group. This shows the need for more investment in research and the development of new treatment strategies against this pathogen [10]. Here we present a rare case of a patient with prosthetic valve endocarditis caused by *N. glabrata* and illustrate a therapeutic dilemma because of the limited (surgical and antifungal) treatment options and clinical research data.

## 2 Case

**Fig. 1:**
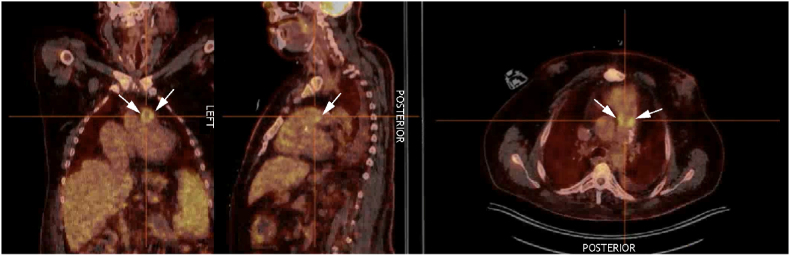
PET-CT scan showing FDG uptake in the pulmonary prosthetic valve (white arrows) suggestive of endocarditis.

A 47-year-old male patient with a surgically corrected Tetralogy of Fallot and pulmonary valve replacement (BioPulmonic Conduit) was admitted to the cardiology ward for treatment of symptomatic right heart failure (day 0). This was caused by progressive right ventricular dilatation and dysfunction secondary to severe supravalvular stenosis of his pulmonary bioprosthesis. His medical history reports multiple co-morbidities including recurrent supraventricular tachycardia, diabetes mellitus type I, obesity and intellectual disability. No history of IV drug use or recent immunosuppressive therapy was reported. On admission, he received IV diuretic treatment and a percutaneous intervention was planned to treat the supravalvular pulmonary stenosis. However, on day +11 after admission he developed fever (40.5 °C) with elevated C-reactive protein (237.9 mg/L) and leukocytosis (14.0 × 10^9/L). Blood cultures taken at day +11 showed growth of *N. glabrata*, susceptible to only anidulafungin (MIC 0.016 mg/L) and Amphotericin B (MIC 0.38 mg/L) as determined by antifungal susceptibility testing using the EUCAST reference method. Anidulafungin IV 200 mg loading dose followed by 100 mg IV daily was started at day +13 and follow-up blood cultures were taken routinely. Transthoracic echocardiography on day +15 showed poor right ventricular function, severe pulmonary valve stenosis, and severe tricuspid regurgitation, but no signs of valve vegetation. A PET-CT scan to evaluate disseminated disease at day +23 (Fig. 1) showed increased FDG uptake in the pulmonary prosthetic valve, and the diagnosis *N. glabrata* prosthetic valve infective endocarditis was made. Ophthalmic examination showed no signs of endogenous chorioretinitis or endophthalmitis. At day +14, after 1 day of treatment with IV anidulafungin, the patient improved clinically with decreasing inflammation markers in the blood and resolution of fever. However, the blood cultures were persistently positive for 35 days, albeit with periods of negative cultures, despite adequate treatment with anidulafungin IV 100 mg daily initially and after increasing anidulafungin IV to 200 mg daily at day +30. International guidelines recommend early surgery to treat *Candida* prosthetic valve infective endocarditis or if surgery is not possible, IV treatment with liposomal Amphotericin B with or without flucytosine or high-dose echinocandin followed by long-term suppressive therapy with fluconazole [[11], [12], [13]]. In our patient, surgical intervention was already indicated because of his severe supravalvular pulmonary stenosis causing poor right ventricular systolic function, regardless of the presence of endocarditis. However, due to the high perioperative risk, a multidisciplinary team decided, in consultation with the patient and his family, that the high risk of complications outweighed the potential benefits of surgery. Therefore, long-term suppressive therapy with an echinocandin and/or liposomal Amphotericin B via IV route, with potential adverse effects such as toxicity, metastatic abscesses and relapse, was the only remaining treatment option. We decided to switch to liposomal Amphotericin B IV 3 mg/kg daily monotherapy instead of double therapy with flucytosine at day +46 because of a nationwide shortage of flucytosine. Eventually, the last positive blood cultures were taken at day +46 and remained sterile during follow-up, possibly after switching to liposomal Amphotericin B IV. The patient was discharged at day +74 and referred to outpatient parenteral antimicrobial therapy (OPAT) to complete the treatment course of 6 weeks in total, followed by long-term suppressive therapy with anidulafungin IV 100 mg daily at day +88. During follow-ups, the inflammatory markers remained at a low level and no signs of antifungal treatment failure were observed for over 1 year, up till the patient's death due to progressive right ventricular failure at day +411.

## 3 Discussion

The increasing incidence of invasive fungal infections and antifungal resistance is an emerging threat to global health. Moreover, the limited arsenal of antifungal drugs in combination with a scarcity of high-quality clinical data can make patients with invasive fungal infections difficult to treat [10,14]. Here we report a case of prosthetic valve infective endocarditis in which choosing the optimal treatment was challenging because of the intrinsic antifungal resistance of *N. glabrata* in combination with the patient's comorbidity and the limited evidence base for treatment of *N. glabrata* endocarditis.

Current international guidelines for the treatment of *Candida* prosthetic valve endocarditis recommend early surgery followed and long-term suppressive therapy with fluconazole [11,12]. However, surgery was not viable for our patient because of the high perioperative risks due to his comorbidities. Therefore, pharmacological treatment with antifungals was initiated, which is the second choice according to the guidelines.

Additional treatment with fluconazole was not possible due to the intrinsic resistance of *N. glabrata* against azoles. It is important to note though, that these recommendations are based on small case studies and expert opinions [2,3,12,15]. Moreover, it remains unclear whether early surgical intervention is indeed more beneficial as compared to singular IV antifungal therapy [16,17].

Fortunately, the patient tolerated IV anidulafungin well without signs of toxicity, metastatic abscesses or relapse, despite the long treatment duration. We are however aware of the possible emergence of echinocandin resistance of *N. glabrata* that has been documented in patients with prolonged treatment [18]. In the case of relapse with an echinocandin-resistant *N. glabrata*, there are no recommendations for an alternative in the aforementioned guidelines and other available clinical evidence is limited [11,12]. Therefore, the development of new antifungals and more clinical studies are urgently needed. Recently, some progress has been made with promising new drug candidates for the treatment of invasive fungal infections, such as Fosmanogepix and Rezafungin [19,20]. Fosmanogepix is a Gwt1 enzyme inhibitor with a novel mechanism of action and good (oral) bioavailability [19]. Rezafungin is a long-acting second-generation echinocandin with good bioactivity that can be administered once a week (IV), which is a more patient-friendly option for long-term suppressive therapy [20]. Both drugs show promising broad-spectrum activity against fungal pathogens, including *N. glabrata*, and are effective against biofilm formation, which is important in the treatment of (fungal) infective endocarditis [19,20]. These drugs are therefore potential alternatives for our patient when available in the future.

In conclusion, this case report illustrates the therapeutic challenges of prosthetic valve endocarditis by a drug-resistant fungal pathogen in a patient with substantial comorbidity. With the alarming increase in resistance to antifungals, the discovery and development of novel antifungals is urgently needed. Moreover, more clinical studies, in particular RCTs, are necessary to optimize the management of endocarditis caused by drug-resistant fungi.

## Ethical form

Please note that this journal requires full disclosure of all sources of funding and potential conflicts of interest. The journal also requires a declaration that the author(s) have obtained written and signed consent to publish the case report from the patient or legal guardian(s).

The statements on funding, conflict of interest and consent need to be submitted via our Ethical Form that can be downloaded from the submission site www.ees.elsevier.com/mmcr. Please note that your manuscript will not be considered for publication until the signed Ethical Form has been received.

## Funding source

There are none.

## Consent

Written informed consent was obtained from the patient or legal guardian(s) for publication of this case report and accompanying images. A copy of the written consent is available for review by the Editor-in-Chief of this journal on request.

## Declaration of competing interest

There are none.
